# A life dedicated to science, teaching and leadership: Professor Emeritus Marcos Felipe Silva de Sá

**DOI:** 10.61622/rbgo/2026rbgoedt5

**Published:** 2026-10-20

**Affiliations:** 1 Universidade de São Paulo Faculdade de Medicina de Ribeirão Preto Ribeirão Preto SP Brazil Faculdade de Medicina de Ribeirão Preto, Universidade de São Paulo, Ribeirão Preto, SP, Brazil; 2 Universidade Federal de Minas Gerais Belo Horizonte MG Brazil Universidade Federal de Minas Gerais, Belo Horizonte, MG, Brazil; 3 Universidade Federal do Rio Grande do Sul Porto Alegre RS Brazil Universidade Federal do Rio Grande do Sul, Porto Alegre, RS, Brazil

**Figure:**
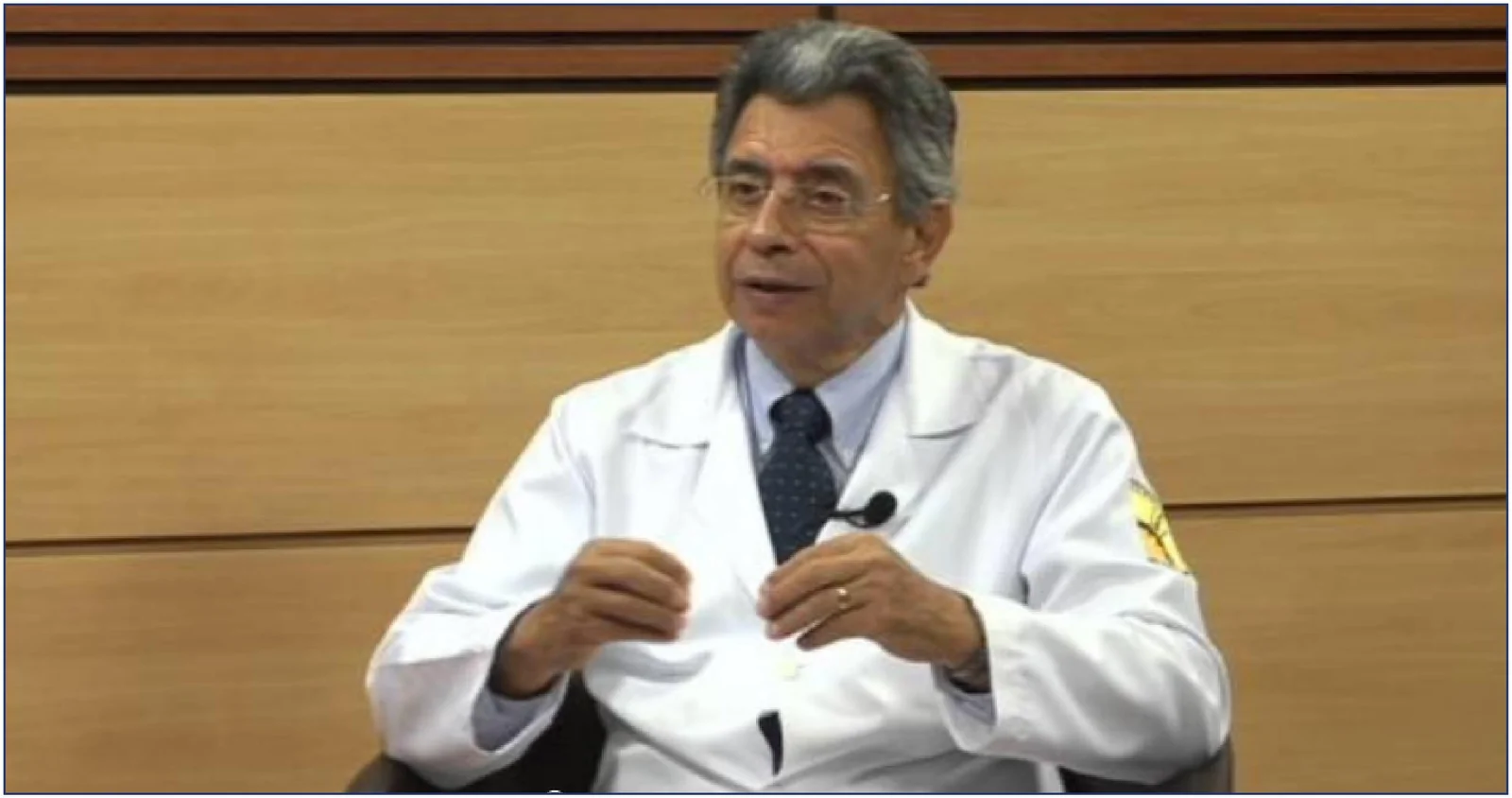
Marcos Felipe Silva de Sá - Faculdade de Medicina de Ribeirão Preto at the Universidade de São Paulo (FMRP-USP)

Skillfully intertwined with the history of Brazilian Gynecology and Obstetrics and the strengthening of our academic institutions, Professor Marcos Felipe Silva de Sá has reached a definitive milestone in his career: the conferral of the title of Professor Emeritus by the Faculdade de Medicina de Ribeirão Preto at the Universidade de São Paulo (FMRP-USP). As he serves as the current Editor-in-Chief of our *Revista Brasileira de Ginecologia e Obstetrícia* (RBGO), this honor transcends the boundaries of the Ribeirão Preto university campus, resonating within the hearts of the entire national and international scientific community dedicated to women's health.

Born in Guaxupé, Minas Gerais, in 1947, Professor Marcos Felipe began his academic career at FMRP-USP on October 30, 1973. Over more than five decades of uninterrupted service, he built a career defined by exemplary dedication, methodological rigor, and a transformative institutional vision. A medical graduate of FMRP-USP, he completed his Master's and Doctoral degrees in Obstetrics and Gynecology at the same institution, eventually reaching the pinnacle of his university career by becoming a Full Professor in the Department of Obstetrics and Gynecology in 1990.

His constant pursuit of cutting-edge knowledge led him to undertake a distinguished postdoctoral fellowship in the Department of Reproductive Medicine at the University of California, San Diego (UCSD), working alongside prominent figures such as Professor Samuel Sheng-Tsai Yen and Professor Robert William Rebar, as well as specialized training in Human Reproduction at the University of Valencia, Spain, under Professors Antonio Pellicer and Fernando Bonilla-Musoles. These international experiences early on solidified his global standing and influence in the fields of Endocrine Gynecology and Reproductive Medicine.

## Scientific legacy and academic mentorship in numbers

In the field of scientific research, the output of Professor Marcos Felipe Silva de Sá exemplifies a tireless dedication to generating knowledge and advancing new scientific concepts. He was a CNPq Level 1 researcher, producing work in the field of Reproductive Medicine with an emphasis on Endocrine Gynecology and the climacteric from 1987 to 2018, and participated in major scientific initiatives such as the National Institute of Science and Technology (Instituto Nacional de Ciência e Tecnologia, INCT) for Hormones and Women's Health. He has authored an extensive body of work comprising over 460 articles published in high-impact national and international journals, as well as 11 books and 59 book chapters that have become reference works for the specialty in Brazil.

His studies address crucial topics in female reproductive physiology and pathophysiology:

- Physiology and regulatory mechanisms of the menstrual cycle
- Endocrinology of the climacteric and hormone replacement therapy
- Polycystic Ovary Syndrome (PCOS) and hyperandrogenic states
- Hormonal markers and metabolism during the menopausal transition

The most profound legacy of a public university faculty member lies in the perpetuation of knowledge through the minds they shape. Throughout his career in USP's graduate programs and the Professional Master's Program in Health Organization Management, Professor Marcos Felipe advised 24 doctoral dissertations and 33 master's theses, in addition to overseeing postdoctoral research and undergraduate research projects. His former students now hold leadership positions in universities, research centers, and hospitals across Brazil and abroad, carrying forward the ethical stance, investigative discipline, and humanism that the mentor always instilled in the practice of medical education.

## A public calling and mastery of institutional management

Few top-tier researchers manage to combine investigative excellence with an administrative calling and management capabilities of the highest caliber. Professor Marcos Felipe Silva de Sá played pivotal roles in shaping the administrative and clinical care structures of the Universidade de São Paulo and its hospital complex.

Key academic and hospital leadership positions he held include:

- Head of the Department of Gynecology and Obstetrics at FMRP-USP (1991–1993);
- Dean of the Faculdade de Medicina de Ribeirão Preto at the Universidade de São Paulo (2005–2009);
- Superintendent of the Hospital das Clínicas of FMRP-USP across two separate terms (1995–2003 and 2011–2015), leading structural expansions and the enhancement of high-complexity care within the Unified Health System (SUS);
- Chief Administrative Officer of the Rector's Office at the Universidade de São Paulo (2009–2010);
- First Executive Director and Founder of the Foundation for the Support of Teaching, Research, and Care (FAEPA, Fundação de Apoio ao Ensino, Pesquisa e Assistência) (1988–1992), an organization where he also chaired the Board of Trustees;
- President of the Fundação de Pesquisas Médicas de Ribeirão Preto (FUPEME).

To provide a scientific foundation for his management practices and ensure that his administrative approach aligned with modern best practices, the honoree completed an MBA in Health Management in 2004 at the Faculdade de Economia, Administração e Contabilidade de Ribeirão Preto (FEARP-USP). The achievements resulting from his management work left a lasting legacy at the major institutions where he served. A particular highlight was his role in implementing and consolidating the SUS within the HCRP, integrating it into a vast healthcare complex, surely one of the largest in the country's interior.

## Involvement in professional societies and international engagement

Professor Marcos Felipe's leadership extended beyond the university setting, profoundly influencing professional associations and the regulation of medical practice in Brazil. A pioneer in establishing the field of Endocrine Gynecology in the country, he played an active role in organizing this medical specialty:

1. Founder and first President of the Brazilian Society of Endocrine Gynecology (Sociedade Brasileira de Ginecologia Endócrina, SOBRAGE);
2. Founder of the Brazilian Climacteric Association (Associação Brasileira de Climatério, SOBRAC);
3. Scientific Coordinator for Gynecology at the Obstetrics and Gynecology Association of the State of São Paulo (Associação de Obstetrícia e Ginecologia do Estado de São Paulo, SOGESP);
4. Scientific Director, active member, and director of various specialized national committees within the Brazilian Federation of Gynecology and Obstetrics Associations (FEBRASGO).

Through SOGESP and FEBRASGO, Professor Marcos Felipe played a decisive role in developing consensus statements, clinical guidelines, and practical manuals, as well as promoting continuing medical education programs. His international engagement enabled him to bring prominent global scientific figures to Brazil while simultaneously showcasing the work of Brazilian researchers on continental and global scientific stages, fostering collaborations with the International Menopause Society (IMS) and the International Federation of Gynecology and Obstetrics (FIGO).

## Dedication to scientific editing and impact on the RBGO

Brazilian medical publishing has in Professor Marcos Felipe one of its most enthusiastic advocates and leaders. Upon assuming the role of Editor-in-Chief of the RBGO in January 2016, he brought his extensive experience as an author, reviewer, and consultant for national and international scientific journals to modernize the publication's editorial process.

Under his leadership:

- RBGO consolidated its transition to publishing exclusively in English, expanding its international reach, a move that enabled its indexing and a rising impact factor;
- A rigorous, transparent peer-review system aligned with Open Science principles was implemented;
- Citation metrics showed continuous improvement, and the journal's Qualis/CAPES rating was elevated;
- Maintained a strong commitment to editorial workflow efficiency and the practical relevance of the topics accepted for publication.

In addition to his work with RBGO, Professor Marcos Felipe served on the editorial boards of major scientific journals, such as *Fertility and Sterility*, as well as various advisory boards for national and international publications, acting as a consistent reviewer of key discoveries in the field of obstetrics and gynecology.

## Honoring the master: the title of Professor Emeritus

The awarding of the title of Professor Emeritus by FMRP-USP represents the highest form of institutional recognition a university can bestow upon its faculty. It is reserved for those who have demonstrated remarkable wisdom, exceptional scientific contributions, and exemplary dedication to teaching and the community.

As the honoree himself noted when reflecting on the vocation of teaching:

*"To be an educator is more than just being a teacher. An educator is someone who prepares their mentees for life, taking on the responsibility for developing a range of skills and competencies within them. When entering the classroom, the educator brings not only the content to be taught but also their own life, their way of being, acting, interpreting, and understanding the world."*

The Department of Gynecology and Obstetrics at FMRP-USP, the RBGO, its Editorial Board, and FEBRASGO proudly celebrate this historic moment. We offer our heartfelt tribute to Professor Marcos Felipe Silva de Sá, a brilliant physician, tireless researcher, visionary leader, and unforgettable mentor. May your journey continue to illuminate the present and future generations of Brazilian medicine.

